# A psychometric evaluation of the PedsQL™ Family Impact Module in parents of children with sickle cell disease

**DOI:** 10.1186/1477-7525-7-32

**Published:** 2009-04-16

**Authors:** Julie A Panepinto, Raymond G Hoffmann, Nicholas M Pajewski

**Affiliations:** 1 Department of Pediatrics, The Children's Research Institute of the Children's Hospital of Wisconsin/Medical College of Wisconsin, Milwaukee, WI, USA; 2 Hematology/Oncology/Bone Marrow Transplantation, Medical College of Wisconsin, Milwaukee, WI, USA; 3 Quantitative Health Sciences, Medical College of Wisconsin, Milwaukee, WI, USA; 4 Section on Statistical Genetics, Department of Biostatistics, University of Alabama at Birmingham, Birmingham, AL, USA

## Abstract

**Background:**

Caring for a child with a chronic condition, such as sickle cell disease, can have a significant impact on parents and families. In order to provide comprehensive care and support to these families, psychometrically sound instruments are needed as an initial step in measuring the impact of chronic diseases on parents and families. We sought to evaluate the psychometric properties of the PedsQL™ Family Impact Module in populations of children with and without sickle cell disease. In addition, we sought to determine the correlation between parent's well being and their proxy report of their child's health-related quality of life (HRQL).

**Methods:**

We conducted a cross-sectional study of parents of children with and without sickle cell disease who presented to an urban hospital-based sickle cell disease clinic and an urban primary care clinic. We assessed the HRQL and family functioning of both groups of parents utilizing the PedsQL™ Family Impact Module. The reliability, validity and factor structure of the instrument were determined and scores from the instrument were correlated with scores from parent-proxy report of their child's HRQL using the PedsQL™ 4.0 Generic Core Scales.

**Results:**

Parents of 170 children completed the module (97 parents of children with sickle cell disease and 73 parents of children without sickle cell disease). The Family Impact Module had high ceiling effects but was reliable (Cronbach's alpha > 0.80 in all scales). The empirical factor structure was generally consistent with the theoretical factor structure and supported construct validity. The Family Impact Module discriminated between parents of children with severe sickle cell disease from parents of children with mild disease or no disease in the areas of communication and worry. There were no significant differences across any of the subscales between parents of children with mild sickle cell disease and those with no disease. Parents with higher scores, representing better HRQL and family functioning, generally reported higher HRQL scores for their children.

**Conclusion:**

The PedsQL™ Family Impact module was reliable, however it displayed large ceiling effects and did not discriminate well between parents of children with and without sickle cell disease. Future research to evaluate the psychometric properties of the Family Impact Module for parents of healthy children may be helpful.

## Background

Understanding the impact of a chronic disease on a parent and family of a child with a chronic disease is critical to providing comprehensive care to these families. However, the relationship between a child's disease and its course and the impact on a parent and family is complex and dynamic. Prior research has shown that caregivers with children who have a chronic disease experience stress[1], may have greater family burden[2] and need for social support[3], and provide more caregiver time with their children[4]. In addition, a negative parental perception of a child's health is associated with higher healthcare utilization by the child. [5,6]

Sickle cell disease is a genetic disease usually diagnosed at birth by newborn screening. It is characterized most commonly by frequent, episodic vaso-occlusive painful events that often result in hospitalizations and missed school by the child and work by the parent. [7,8] In addition, children with sickle cell disease experience central nervous system complications such as silent and overt stroke potentially leading to neuro-cognitive deficits. [9,10] Caring for a child with sickle cell disease is thus often met with unpredictability and family burden due to these complications. Only one prior study has examined the impact of sickle cell disease on the health-related quality of life (HRQL) of parents of children with sickle cell disease [6]. They found that female caregivers of children with sickle cell disease displayed lower scores on the depressive moods, daily activities and vitality subscales of the TNO-AZL Adult Quality of Life questionnaire [7] compared to socio-economic matched controls. However, this study was in a Dutch (although primarily immigrant) population and so it is unclear whether these results translate to caregivers of children with sickle cell disease in the United States. In addition, it is not known whether there is an association of a parent's HRQL on the proxy-reporting of their child's HRQL in children with sickle cell disease. In a study of healthy children, parents' HRQL was shown to correlate with their proxy reporting of the child's HRQL. [11]

We sought to determine the psychometric properties of the PedsQL™ Family Impact Module, an instrument designed to assess the impact of chronic disease on parents and families by examining family functioning and parent HRQL. In addition, we also compared parent HRQL with parent-proxy reporting of the child's HRQL in this population. We expected the PedsQL™ Family Impact Module to be valid and reliable. We hypothesized that parents of children with sickle cell disease would have worse HRQL and family functioning than parents of children without sickle cell disease. In addition, we hypothesized that parents with worse HRQL would report that their child has worse HRQL.

## Methods

### Study Setting and Subjects

We conducted a cross sectional study of parents of children with and without sickle cell disease from January 1, 2006 through June, 2007. Study subjects were eligible if: 1) they had a child ages 2 to 18 years of age with sickle cell disease who they accompanied to the Midwest Sickle Cell Center for a routine check up, or 2) they had a child ages 2 to 18 years of age who they accompanied for a routine check-up at the Downtown Health Center in Milwaukee, Wisconsin. Children were excluded from the study if they had an acute illness or were hospitalized within the last month.

The Midwest Sickle Cell Center serves over 300 children with sickle cell disease and is based within an academic children's hospital. The Downtown Health Center is an urban based clinic that provides primary care to over 4,000 children a year. The majority of patients who regularly attend this clinic are African American (80%) and have public insurance (92% Medicaid) therefore representing a similar socio-demographic population to our families with sickle cell disease. In addition, both the Sickle Cell Center and the Downtown Health Center serve patients living in similar zip code areas in the city of Milwaukee and thus are comparable in regards to socio-demographic factors.

Demographic data on the parents and the children were self reported by the parents and collected through medical record review. Race data was collected using a modified United States Census classification and reflect parent report based on the following choices: White, Black, Native Hawaiian or Other Pacific Islander, Asian, American Indian or Alaskan native, Other or Unknown.

Parents were asked to report whether they had ever been told by a health care provider that their child had any of the following medical conditions: Asthma, chronic allergies/sinus trouble, chronic orthopedic/bone/joint problems, chronic rheumatic disease, diabetes, epilepsy, or other chronic medical condition. Patients were classified as having a medical co-morbidity if they reported one or more of the above chronic medical conditions.

In addition, parents were asked to report whether they had ever been told by a health care provider that their child had any of the following neurobehavioral conditions: anxiety, attentional, or behavioral problems, depression, developmental delay or mental retardation, learning problems, or speech problems. Patients were classified as having a neurobehavioral co-morbidity if they reported one or more of the above noted neurobehavioral conditions.

Lastly, to aid in determining discriminant validity of the PedsQL™ Family Impact Module in our population of children with sickle cell disease, we collected data on the disease status of the children with sickle cell disease. Disease status was classified a priori as mild or severe disease regardless of the child's sickle cell genotype. Severe disease was defined as those children with a history of a sickle cell related stroke, acute chest syndrome, 3 or more hospitalizations in the prior 3 years, or recurrent priapism based on criteria used for interventions such as hydroxyurea or bone marrow transplantation and consistent with our prior work in determining disease severity. [12-16] All others were classified as having mild disease.

The Institutional Review Board of the Children's Hospital of Wisconsin/Medical College of Wisconsin approved the study and informed consent was obtained from the parents.

### Outcome Measures

The primary study outcomes were parent HRQL and family functioning as measured by the PedsQL™ Family Impact Module which the parents completed during their child's health care clinic visit.

#### PedsQL™ Family Impact Module

The PedsQL™ Family Impact Module [17] assesses parent HRQL and family functioning and is intended to measure the impact of a chronic health condition on parents and families. It has been shown to be reliable and valid in smaller studies of children with complex special health care needs and children with cancer. [17,18] There are no other studies related to the psychometric properties or development of this instrument to date. The PedsQL™ Family Impact Module consists of 36 items (see last tables for the items included in the module) that make up 8 scales: physical functioning, emotional functioning, social functioning, cognitive functioning, communication, worry, daily activities, and family relationships. Each item is scored on a 5 point response scale which is then transformed to a 0 to 100 scale, with higher scores denoting better parent HRQL and family functioning. Mean scores are then computed by averaging the individual item scores within a particular subscale.

The Family Impact Module yields 3 summary scores: Total score, parent health-related quality of life summary score, and family functioning summary score. The total score is comprised of the average of the responses to all items in the questionnaire. The parent HRQL summary score is determined by averaging the responses to the 20 items that make up the physical, emotional, social and cognitive functioning scales noted above. The family functioning summary score is determined by summing and averaging the responses to the 8 items that make up the daily activities and family relationships scales. Missing items were handled according to the developer's guidelines. [17]

#### PedsQL™ 4.0 Generic Core Scales

In addition to the PedsQL™ Family Impact Module, each parent also completed the parent proxy-report of the PedsQL™ 4.0 Generic Core Scales. The PedsQL™ is a 23 item generic HRQL questionnaire with a proxy report for children ages 2 through 18 years. [19] The questionnaire takes 5 to 10 minutes to complete. The questionnaire yields information on the physical, emotional, social and school functioning of the child during the previous 4 weeks. Mean scores are calculated based on a 5-point response scale for each item and transformed to a 0 to 100 scale with a higher score representing better quality of life. The PedsQL™ yields 3 summary scores: a total scale score, a physical health summary score, and a psychosocial health summary score. There are 4 scale scores: physical functioning, emotional functioning, social functioning, and school functioning. The total score is comprised of the average of all items in the questionnaire. The psychosocial summary score is comprised of the average of the items in the emotional, social, and school functioning scales. The physical health summary score is comprised of the average of items in the physical functioning scale and is the same score as the physical functioning score. Missing items were accounted for based on the developer's recommendation. [19]

The psychometric properties of the PedsQL™ Generic Core Scales have been extensively studied within populations with a wide array of chronic health conditions, including sickle cell disease. [13-20] In contrast, the properties of the PedsQL™ Family Impact Module have only been demonstrated within populations of children with special health care needs and cancer. [17,18] Therefore it is unclear whether the Family Impact Module is a valid and reliable measure for assessing the impact of SCD on parents and families. We therefore analyzed the following properties of the PedsQL™ Family Impact Module within our population of parents, both with and without sickle cell disease.

### Statistical Analysis

Descriptive statistics were calculated for parent and child characteristics. Categorical variables are presented as observed frequencies and proportions. Comparisons of categorical factors were performed using Chi-Square Tests or Fisher's Exact/Fisher-Freeman-Halton Tests where appropriate. Mean scores for HRQL were calculated using the PedsQL™ developer's guidelines. [20] Effect sizes, calculated by taking the differences between means and dividing by the pooled standard deviation, were done to show the magnitude of the differences between parents of children with and without sickle cell disease. Based on standard accepted criteria, effects sizes were considered small (0.2), medium (0.5) and large (0.8). [21]

### Floor/Ceiling Effects

The percentage of scores on the PedsQL™ Family Impact Module that were at the ceiling (top of the scale) or floor (bottom of the scale) were calculated for each subscale and summary score. A percentage less than 25% was considered a low ceiling/floor effect. [22,23]

### Reliability and Validity

We assessed the internal consistency reliability of the PedsQL™ Family Impact Module to determine whether the items within each scale were consistent with each other. This was assessed using Cronbach's alpha for each of the 8 subscales of the PedsQL™ Family Impact Module as well as for the summary and total scores. A Cronbach's alpha coefficient of greater than 0.70 was considered acceptable for group-level analysis. [24]

Validity was determined using a known-groups comparison method. To determine the discriminant validity of the PedsQL™ Family Impact Module, comparisons were made between parents of children with and without sickle cell disease. In addition, we also compared parents of children with sickle cell disease to parents of children without sickle cell disease and without other co-morbidities to ensure a comparison to "healthy" children. Because of skewed distributions, mean summary and subscale scores for the parents of children with and without sickle cell disease are reported as medians and interquartile ranges (IQR). Comparisons of summary and subscale scores were made using non-parametric Wilcoxon or Kruskal-Wallis tests.

To further determine the validity of the PedsQL™ Family Impact Module, an exploratory factor analysis was performed to determine if items correlated as expected for the scale structure. The factor analysis was based on the polychoric correlation matrix due to the ordinal nature of the module items. Extracted factors were based on the eigenvalue > 1.0 criterion and were rotated using the promax oblique rotation. All analyses were performed using SAS v9.1.3 (SAS, Cary, NC).

### Correlation Between Family Impact Module and Parent-proxy PedsQL™ Health-related Quality of Life report

To determine the association between the parent's HRQL and family functioning with the proxy-report of their child's HRQL, we examined the correlation between the scores on the PedsQL™ Family Impact Module with corresponding parent-proxy PedsQL™ HRQL scores. Spearman rank correlations were used to correlate the summary and subscale scores from the Family Impact Module with parent-proxy reports of the child's HRQL based on the PedsQL™ Generic Core Scales. Coefficients less than 0.3 in absolute magnitude were considered indicative of weak correlation, between 0.3 and 0.5 moderate correlation, and greater than 0.5 as strong correlation. [21]

## Results

We recruited a convenience sample of parents of children with and without sickle cell disease at both clinics as part of a larger study of the HRQL of children. [13,25] There were 145 parents of children with sickle cell disease approached for the study. Of those, 133 agreed to participate and 20 refused for varying reasons such as "not enough time". Ninety-seven parents of children with sickle cell disease completed both a PedsQL™ Family Impact module and the generic PedsQL™ parent proxy report. Ninety-four parents of children without sickle cell disease were approached at the Downtown Health Center. Of those, 74 control parents agreed to participate and 73 completed both a PedsQL™ Family Impact module and generic PedsQL™ parent proxy report.

The majority of respondents were female, the biological parent and not married (Table 1). Parents of children with sickle cell disease were older, had higher income, and education than parents of children without sickle cell disease. The majority of parents in each group were African American. A significant number of children from both groups had other co-morbidities. Slightly more than half of the children with sickle cell disease were classified as having severe disease.

**Table 1:** Demographic Characteristics of the Sample

	Parents of SCD children (N = 97)	Parents of Control children (N = 73)	p-value
**Characteristics of the Parents**			
			
**Age (yrs)**, median (P25, P75)	37.1 (31.1,40.9)	29.2 (25.9, 35.0)	<0.001
**Gender**	N (%)	N (%)	
Male	7 (7.2)	6 (8.2)	0.967
Female	89 (91.8)	66 (90.4)	
Not Reported	1 (1.0)	1 (1.4)	
			
**Type of Caregiver**			
Biological parent	85 (87.6)	55 (75.3)	0.681
Step parent	0 (0.0)	1 (1.4)	
Foster parent	0 (0.0)	0 (0.0)	
Adoptive parent	2 (2.1)	1 (1.4)	
Guardian	6 (6.2)	7 (9.6)	
Other	2 (2.1)	1 (1.4)	
Not Reported	2 (2.1)	8 (11.0)	
			
**Race/Ethnicity**			
African-American	60 (61.9)	50 (68.5)	0.010
Other	3 (3.1)	14 (19.2)	
Not Reported	34 (35.1)	9 (12.3)	
			
			
**Family Income**			
< $20 K	37 (38.1)	34 (46.6)	0.032
≥ $20 K but <$40 K	24 (24.7)	13 (17.8)	
≥ $40 K	21 (21.7)	5 (6.9)	
Not Reported	15 (15.5)	21 (28.8)	
			
**Marital Status**			
Married	23 (23.7)	21 (28.8)	0.604
Not Currently Married	62 (62.9)	44 (60.3)	
Not Reported	12 (12.4)	8 (11.0)	
			
**Highest Education Level**			
Some High School or less	20 (20.6)	28 (38.4)	0.0315
High School diploma/GED	29 (29.9)	12 (16.4)	
Vocational/some college	31 (32.0)	24 (32.9)	
College/graduate degree	8 (8.3)	3 (4.1)	
Not Reported	9 (9.3)	6 (8.2)	
			
**Work Status**			
Working	56 (57.7)	10 (13.7)	<0.001
Not Working	31 (32.0)	56 (76.7)	
Not Reported	10 (10.3)	7 (9.6)	
			
**Characteristics of Children**			
			
**Age (yrs)**, median (P25, P75)	10.0 (5.0, 14.0)	7.0 (3.0, 11.0)	0.005
			
**Gender**			
Female	52 (53.6)	41 (56.2)	0.740
			
**Medical Co-morbidities**			
Any	27 (27.8)	34 (46.6)	0.012
Asthma	20 (20.6)	27 (37.0)	
Chronic Allergies	6 (6.2)	11 (15.1)	
Diabetes	1 (1.0)	1 (1.4)	
Chronic Orthopaedic, Bone, or Joint Problems	8 (8.3)	3 (4.1)	
Epilepsy	4 (4.1)	1 (1.4)	
Rheumatic Disease	1 (1.0)	0 (0.0)	
Other	2 (2.1)	4 (5.5)	
			
**Neuro-behavioral Co-morbidities**			
Any	38 (39.2)	34 (46.6)	0.334
Anxiety Problems	6 (6.2)	9 (12.3)	
Attentional Problems	22 (22.7)	17 (23.3)	
Behavioral Problems	19 (19.6)	17 (23.3)	
Depression	7 (7.2)	6 (8.2)	
Developmental Delay or Mental Retardation	4 (36.4)	7 (63.6)	
Learning Problems	21 (21.7)	14 (19.2)	
Speech Problems	9 (9.3)	10 (13.7)	
			
**Sickle Cell Disease Status**			
Mild disease	44 (45.5)	NA	
Severe disease	53 (54.6)	NA	

P25 and P75: 25% and 75% percentile

### Floor/Ceiling Effects in Children with Sickle Cell Disease

The PedsQL™ Family Impact Module demonstrated low floor effects in all summary and scale scores for both groups (Table 2). However, moderate to high ceiling effects were observed for the emotional functioning, social functioning, cognitive functioning, communication, daily activities and family relationships subscales. In parents of children without sickle cell disease, there was also a moderate ceiling effect in the family functioning summary scale and the worry scale.

**Table 2:** Scale Descriptives of the PedsQL™ 2.0 Family Impact Module in Children with Sickle Cell Disease and Urban Controls

	**Sickle Cell Disease**			**Controls**				
Scale	N	Median (IQR)	%Floor/%Ceiling	N	Median (IQR)	%Floor/%Ceiling	Effect Size	p-value*
Total Score	95	75.7 (60.8, 86.8)	0.0/4.2	73	75.7 (62.5, 91.3)	0.0/6.8	-0.061	0.549
Parent HRQL	97	75.0 (61.3, 90.0)	0.0/6.2	72	74.4 (59.7, 90.0)	0.0/9.7	0.052	0.793
Family Functioning	94	75.0 (62.5, 93.8)	1.1/19.1	68	81.3 (63.1, 100.0)	0.0/27.9	-0.072	0.414
								
Physical Functioning	95	66.7 (50.0, 87.5)	1.1/13.7	72	70.8 (54.2, 91.7)	0.0/18.1	-0.139	0.374
Emotional Functioning	97	75.0 (60.0, 100.0)	0.0/26.8	73	75.0 (60.0, 100.0)	0.0/27.4	0.022	0.763
Social Functioning	96	87.5 (62.5, 100.0)	1.0/42.7	72	81.3 (60.9, 100.0)	0.0/37.5	0.133	0.337
Cognitive Functioning	96	75.0 (60.0, 100.0)	1.0/30.2	72	75.0 (53.8, 96.3)	0.0/25.0	0.194	0.234
								
Communication	94	83.3 (66.7, 100.0)	1.1/39.4	72	91.7 (75.0, 100.0)	1.4/47.2	-0.221	0.108
Worry	94	65.0 (45.0, 90.0)	1.1/18.1	71	80.0 (52.5, 100.0)	0.0/33.8	-0.275	0.040
								
Daily Activities	93	75.0 (50.0, 100.0)	3.2/28.0	69	75.0 (50.0, 100.0)	0.0/40.6	-0.120	0.431
Family Relationships	94	80.0 (61.3, 100.0)	1.1/34.0	68	87.5 (65.0, 100.0)	1.5/33.8	-0.015	0.869

*p-value from Wilcoxon Rank-Sum Test

IQR: Inter-quartile Range

### Reliability and Validity

The PedsQL™ Family Impact Module demonstrated acceptable reliability in all summary scores and all scale scores in both groups of parents as evidenced by a Cronbach's alpha coefficients greater than 0.8 (Table 3). When we compared scores between children with and without sickle cell disease as a whole, the groups differed only in the subscale of worry and there were mild effect sizes noted in this subscale and the communication subscale (Table 2). In addition, when we looked for differences in scores between children 12 years and younger and those older than 12 years, we did not see any further differences between groups (data not shown).

**Table 3:** Internal Consistency Reliability of the PedsQL™ Family Impact Module in Children with Sickle Cell Disease and Urban Controls

Scale	Total Sample	Sickle Cell Disease Sample	Control Sample
Total Score	0.96	0.96	0.97
Parent HRQL Summary Score	0.95	0.94	0.95
Family Functioning Summary Score	0.92	0.92	0.93
			
Physical Functioning	0.90	0.89	0.92
Emotional Functioning	0.85	0.86	0.86
Social Functioning	0.83	0.86	0.81
Cognitive Functioning	0.92	0.94	0.91
Communication	0.80	0.81	0.80
Worry	0.89	0.89	0.89
Daily Activities	0.86	0.88	0.84
Family Relationships	0.94	0.95	0.92

Values denote Cronbach's α coefficient

When we compared scores of parents of children with mild sickle cell disease or severe sickle cell disease to parents of children without sickle cell disease, there were significant differences in the communication and worry scales of the module with mild or moderate effect sizes noted in these subscales and in the daily activities subscale. (Table 4). Specifically, parents of children with severe sickle cell disease reported significantly worse scores in the communication and worry scales than children with mild sickle cell disease and children without sickle cell disease.

**Table 4:** Comparison of Parent Health-related Quality of Life and Family Functioning in Children With Sickle Cell Disease and Urban Controls

	**Controls**^**a**^		**Mild SCD**^**b**^		**Severe SCD**^**c**^		
		
Scale	N	Median (IQR)	N	Median (IQR) Effect Size^†^	N	Median (IQR) Effect Size^†^	Differences*
**Summary Scores**							
Total Score	73	75.7 (62.5, 91.3)	44	78.8 (67.4, 91.3)	51	73.6 (57.4, 83.6)	
		-		0.109		-0.211	
Parent HRQL	72	74.4 (59.4, 90.0)	44	78.8 (65.4, 93.1)	53	73.5 (60.0, 84.7)	
		-		0.168		-0.051	
Family Functioning	68	81.3 (61.8, 100.0)	44	76.6 (68.8, 98.4)	50	75.0 (56.3, 93.8)	
				0.038		-0.165	
**Dimensions**							
Physical Functioning	72	70.8 (54.2, 91.7)	43	66.7 (50.0, 91.7)	52	68.8 (50.0, 77.1)	
				-0.100		-0.173	
Emotional Functioning	73	75.0 (60.0, 100.0)	44	80.0 (70.0, 100.0)	53	70.0 (60.0, 90.0)	
				0.185		-0.111	
Social Functioning	72	81.3 (59.4, 100.0)	44	93.8 (68.8, 100.0)	52	87.5 (59.4, 100.0)	
				0.186		0.085	
Cognitive Functioning	72	75.0 (52.5, 97.5)	44	92.5 (60.0, 100.0)	52	75.0 (55.0, 90.8)	
				0.335		0.066	
Communication	73	91.7 (75.0, 100.0)	44	95.8 (66.7, 100.0)	50	66.7 (58.3, 100.0)	a, b > c
				-0.024		-0.403	
Worry	72	80.0 (50.0, 100.0)	44	77.5 (50.0, 100.0)	50	55.0 (40.0, 80.0)	a, b > c
				0.003		-0.532	
Daily Activities	70	75.0 (50.0, 100.0)	44	75.0 (58.3, 100.0)	49	66.7 (50.0, 91.7)	
				-0.001		-0.225	
Family Relationships	68	87.5 (65.0, 100.0)	44	85.0 (62.5, 100.0)	50	80.0 (60.0, 100.0)	
				0.071		-0.088	

* Differences based on Kruskal-Wallis Tests followed by pair-wise Wilcoxon tests if global null hypothesis was rejected

**Figure 1:**
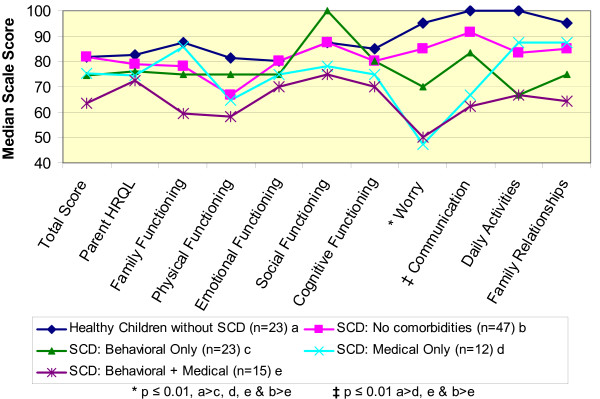
**Median Summary and Scale Scores for PedsQL™ Family Impact Module**. A Comparison of Parents of Healthy Children without Sickle Cell Disease to Children with Sickle Cell Disease.

In our subset analysis (Figure 1) we analyzed the following groups for differences in scores: 1) sickle cell disease without co-morbidities (n = 47), 2) sickle cell disease with neurobehavioral co-morbidities (n = 23), 3) sickle cell disease with medical co-morbidities (n = 12), 4) sickle cell disease with neurobehavioral and medical co-morbidities (n = 15), and 5) healthy, urban controls (n = 23). Children without sickle cell disease and without other co-morbidities tended to have higher scores across most of the subscales and children with sickle cell disease and medical and neurobehavioral co-morbidities tended to have the lowest scores. However, similar to our analysis above, we found statistically significant differences only in the worry and communication subscales.

### Correlation Between Family Impact Module and Parent-proxy PedsQL™ Health-related Quality of Life report

We compared parent proxy HRQL scores for the child[13] to the parent's HRQL scores on the PedsQL™ Family Impact Module and found they were significantly correlated (Table 5). The correlations were moderate for parents of children with sickle cell disease and moderate to high for parents of children without sickle cell disease.

**Table 5:** Spearman Correlations between PedsQL™ Family Impact Module Summary Scores and PedsQL™ Parent-Proxy Report Summary Scores

**Parents of children with SCD**			
	PedsQL™ Family Impact Module		
PedsQL Parent-Proxy Report	Total Score	Parent HRQL	Family Functioning
Total Score	0.456	0.390	0.373
	(<0.001)	(<0.001)	(<0.001)
Psychosocial Health	0.467	0.382	0.403
	(<0.001	(<0.001)	(<0.001)
Physical Health	0.389	0.356	0.278
	(<0.001)	(<0.001)	(0.007)

**Parents of control children**			
	PedsQL™ Family Impact Module		
PedsQL Parent-Proxy Report	Total Score	Parent HRQL	Family Functioning

Total Score	0.603	0.539	0.481
	(<0.001)	(<0.001)	(<0.001)
Psychosocial Health	0.649	0.608	0.649
	(<0.001)	(<0.001)	(<0.001)
Physical Health	0.403	0.320	0.398
	(<0.001)	(0.006)	(0.001)

Number in parentheses denotes p-value for testing H_0_: ρ = 0

### Factor Analysis

Table 6 shows the results of the exploratory factor analysis performed with the PedsQL™ Family Impact Module. Our analysis found 5 factors (latent constructs). Four of the factors generally corresponded to the physical functioning, cognitive functioning, worry, and family relationships scales. However, two social functioning items "feel isolated from others" and "trouble getting support from others", a communication item "feel others don't understand my family's situation", and three emotional functioning items "feel sad", "feel angry", and "feel helpless or hopeless" loaded together on the fifth factor.

**Table 6:** Promax Rotated Factor Loadings for PedsQL™ Family Impact Module

		**Factor**				
	**Item**	I	II	III	IV	V
**Physical Functioning**	...tired during day	0.075	-0.035	**0.932**	0.061	-0.116
	...tired in the morning	0.014	-0.039	**0.942**	0.000	-0.018
	...too tired to do things I like	-0.025	0.078	**0.925**	0.010	-0.034
	...get headaches	-0.018	-0.199	**0.689**	-0.036	0.388
	...feel physically weak	-0.089	0.097	**0.837**	0.210	-0.092
	...feel sick to my stomach	-0.126	-0.087	**0.504**	0.405	0.169

**Emotional Functioning**	...feel anxious	-0.131	0.076	0.306	0.429	0.207
	...feel sad	-0.048	0.057	-0.131	0.244	**0.806**
	...feel angry	-0.043	-0.125	0.074	0.381	**0.677**
	...feel frustrated	0.415	-0.140	0.183	-0.002	0.495
	...feel helpless or hopeless	0.189	0.110	-0.035	-0.036	**0.752**

**Social Functioning**	...feel isolated from others	0.149	0.117	0.191	-0.016	**0.555**
	...trouble getting support from others	0.176	0.188	0.072	0.029	**0.544**
	...hard to find time for social activities	0.138	0.150	0.402	0.011	0.361
	...enough energy for social activities	-0.006	0.246	0.387	0.192	0.256

**Cognitive Functioning**	...hard to keep my attention on things	0.081	0.200	0.192	**0.635**	-0.083
	...hard to remember what people tell me	0.092	-0.006	0.127	**0.825**	0.009
	...hard to remember what I just heard	0.214	0.012	0.026	**0.782**	0.049
	...hard to think quickly	0.073	0.185	-0.095	**0.753**	0.124
	...trouble remembering what I was just thinking	0.201	-0.087	0.070	**0.676**	0.128

**Worry**	...whether child's medical treatments are working	-0.126	**0.850**	0.013	0.041	0.179
	...side effects of my child's medications	-0.120	**0.886**	-0.060	0.205	0.018
	...how others will react to my child's condition	0.051	**0.854**	-0.108	0.285	-0.130
	...my child's illness affects other family members	0.121	**0.767**	0.036	-0.060	0.039
	...about my child's future	0.005	**0.869**	-0.002	-0.162	0.102

**Communication**	...feel others don't understand my families situation	-0.027	0.389	-0.047	-0.073	**0.666**
	...hard to talk about my child's health	-0.107	**0.528**	-0.047	0.145	0.434
	...hard to tell doctors and nurses how I feel	0.167	0.305	-0.021	0.249	0.339

**Family Relationships**	...lack of communication between family members	**0.848**	-0.031	-0.044	0.064	0.099
	...conflicts between family members	**0.911**	-0.027	0.019	0.065	-0.059
	...difficulty making decisions together as a family	**0.890**	0.042	-0.045	0.103	-0.013
	...difficulty solving family problems together	**0.904**	0.103	-0.048	0.077	-0.016
	...stress or tension between family members	**0.930**	-0.188	-0.109	0.138	0.144

**Daily Activities**	...family activities take more time and effort	0.450	**0.512**	0.247	-0.046	-0.174
	...difficulty finding time to finish household tasks	0.490	0.330	0.301	-0.187	0.085
	...feeling too tired to finish household tasks	0.406	0.372	0.219	-0.085	0.131

Total Variance Explained = 78.2%

Highlighted cells denote factor loadings > 0.50

Results based on n = 139 with complete responses to all items

## Discussion

The PedsQL™ Family Impact Module demonstrated good reliability and indicated that parents of children with sickle cell disease may experience more worry and difficulty with communication surrounding issues related to their child's health. The questionnaire did not differentiate parents of children with sickle cell disease from those without disease in any other area. The empirical factor structure deviated some from its theoretical expectation particularly in terms of the emotional and social functioning, daily activities, and communication subscales. The large proportion of ceiling effects across both study groups suggests that the questionnaire may not be sensitive enough for parents of children in our groups.

Only one prior study has examined the HRQL of parents of children with sickle cell disease. [26] Conducted in the Netherlands, this study found that parents of children with sickle cell disease have worse HRQL than Dutch parents of children from a normative population and to a control group that was matched on socioeconomic status. Our parents of children without sickle cell disease were younger and more likely to not be working and have lower family income compared to our parents of children with sickle cell disease. Although a significant number of our children without sickle cell disease had a chronic illness such as asthma, our subgroup analysis of those without co-morbidities did not demonstrate any additional differences between our two groups. Lastly, it has been shown that urban children from poor socioeconomic areas have low HRQL that is similar to the HRQL of children with a chronic disease [27] but there are no similar data on this in the parents of children with chronic disease.

Two prior studies utilizing the PedsQL™ Family Impact Module found significant differences between groups in the parents' HRQL and family functioning. [17,18] In a group of children with complex health conditions, parents who cared for a child at home had significantly worse HRQL and family functioning on the Family Impact module compared to parents whose child resided in a long term convalescent home. [17] A recent study of Brazilian children with cancer reported worse parent HRQL and family functioning in parents whose children were receiving outpatient chemotherapy compared to parents whose children were receiving inpatient chemotherapy. [18] Both of these studies reported much higher effect sizes than demonstrated in our study and demonstrated expected known group differences, namely that caring for a child with a chronic condition and medical needs at home results in worse parent HRQL and worse family functioning. In addition, both of these prior studies compared caregivers of children with similar illnesses in both groups. Our population included a group of parents whose children had sickle cell disease and compared their well being to parents whose children did not have sickle cell disease. All of the children in our study resided in their parents' home.

There are no prior studies that have utilized the PedsQL™ Family Impact Module to determine the HRQL or family functioning of parents who do not have a child with a chronic illness. Therefore, there is unfortunately a current lack of normative data. The parents in our study were drawn from clinics that largely serve patient populations from minority race/ethnic groups and impoverished backgrounds. In addition, the parents may have stressors related to their socioeconomic status which in and of itself places these parents at risk for poor HRQL and decreased family functioning. Lastly, the control group represents families presenting to their primary care doctor for a routine well child check up which may introduce selection bias in that sample. It will therefore be of future interest to determine whether the parents in both our study groups display depressed HRQL and family functioning compared to a normative population. It will also be of interest to investigate the impact of socio-demographic factors on parent HRQL and family functioning as assessed by the PedsQL™ Family Impact Module. Further work utilizing the PedsQL™ Family Impact Module will help clarify this.

We did demonstrate that the HRQL of the parents correlated significantly with the parents'rating of their child's HRQL. That is, when a parent's HRQL was higher, the parent rated their child's HRQL higher. Prior studies have reported similar findings and underscore the importance of understanding a parents' well being when measuring a child's HRQL and the parent is a proxy-reporter. [11,28]

Similar to two prior studies that examined the reliability of the PedsQL™ Family Impact Module in other chronic diseases, we found the questionnaire to be very reliable. [17,18] Our Cronbach's alpha coefficients were all greater than the minimum expected for group level analysis (0.7) and many exceeded the level needed for an individual level analysis (0.9). [29]

We noted a large ceiling effect in many scales of the module. This implies that the module may not be sensitive to parents who are doing well presently and would not be able to demonstrate further improvement in the parents over time in each of these different scale areas. However, we saw little evidence of floor effects, which implies that the measure would be responsive to detecting further deterioration in a parent's HRQL and family functioning. The PedsQL™ Family Impact Module should be investigated within the context of longitudinal studies, both for parents of healthy children and those with chronic health conditions, to evaluate its sensitivity to changes over time.

A number of limitations should be considered in evaluating these study results. The parents were involved in the study during a routine clinic visit and so the reported scores are not in direct response to a disease exacerbation, such as a vaso-occlusive pain event requiring hospitalization. Future research will be required to assess the short term impact of such events on parents and families. In addition, our parents of children without sickle cell disease may have had burdens other than having a child with chronic disease that resulted in our two groups looking similar in parent HRQL and family functioning when all children were included in the analysis. Unfortunately, we were limited by our sample size to explore this issue further. Lastly, our parents came from one geographical area and may not generalize to other regions.

## Conclusion

The PedsQL™ Family Impact Module is a reliable tool and demonstrated that parents of children with sickle cell disease experience more worry and report problems with communication regarding their child's health compared to children without sickle cell disease. The questionnaire did not demonstrate differences between parents of children with and without sickle cell disease in other areas of family functioning and parent HRQL. Further work to establish normal scores on this measure may be helpful in elucidating the true effect of chronic disease in populations of parents who have a child with a chronic disease.

## Abbreviations

HRQL: health-related quality of life

## Competing interests

The authors declare that they have no competing interests.

## Authors' contributions

JP designed study, acquired and interpreted data, and drafted manuscript. RH aided in study design, supervision and interpretation of data analysis, and revised the manuscript. NP aided in study design, analyzed and interpreted the data, and revised the manuscript.
